# Deterministic processes vary during community assembly for ecologically dissimilar taxa

**DOI:** 10.1038/ncomms9444

**Published:** 2015-10-05

**Authors:** Jeff R. Powell, Senani Karunaratne, Colin D. Campbell, Huaiying Yao, Lucinda Robinson, Brajesh K. Singh

**Affiliations:** 1 Hawkesbury Institute for the Environment, University of Western Sydney, Penrith, New South Wales 2751, Australia; 2 The James Hutton Institute, Aberdeen AB15 8QH, Scotland; 3 Department of Soil and Environment, Swedish University of Agricultural Sciences, Uppsala SE-750 07, Sweden; 4 Institute of the Urban Environment, Chinese Academy of Sciences, Xiamen 361021, China; 5 Global Centre for Land-Based Innovation, University of Western Sydney, Penrith, New South Wales 2751, Australia

## Abstract

The continuum hypothesis states that both deterministic and stochastic processes contribute to the assembly of ecological communities. However, the contextual dependency of these processes remains an open question that imposes strong limitations on predictions of community responses to environmental change. Here we measure community and habitat turnover across multiple vertical soil horizons at 183 sites across Scotland for bacteria and fungi, both dominant and functionally vital components of all soils but which differ substantially in their growth habit and dispersal capability. We find that habitat turnover is the primary driver of bacterial community turnover in general, although its importance decreases with increasing isolation and disturbance. Fungal communities, however, exhibit a highly stochastic assembly process, both neutral and non-neutral in nature, largely independent of disturbance. These findings suggest that increased focus on dispersal limitation and biotic interactions are necessary to manage and conserve the key ecosystem services provided by these assemblages.

## Abstract (web summary)

Both deterministic and stochastic processes likely contribute to the assembly of ecological communities. Here, Powell *et al*. measure soil microbial community and habitat turnover across Scotland and show that stochastic processes usually dominate the assembly of fungal but not bacterial communities.

## Graphical abstract

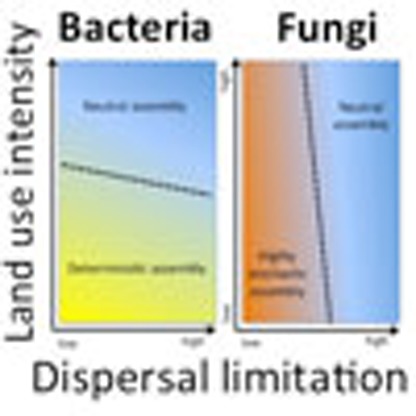

Ecological communities are governed by an interplay of deterministic processes associated with species interactions with their environment (environmental filtering) and each other (limiting similarity)^1^,^2^. In addition, neutral processes associated with dispersal limitation and stochastic demographics within isolated communities also contribute to the outcomes of community assembly^3^. In practice, these deterministic and neutral processes are difficult to separate and both are ultimately governed by the underlying spatial structure of the environment. To tease apart the confounding effects of the overlap among these drivers in the environment, stronger evidence is necessary from studies that directly test the underlying processes of community assembly^1^.

This understanding is relevant for all flora and fauna but not necessarily easily tested in macrobiotic systems due to spatial scaling and long-generation times^4^. The study of microbial communities can overcome these difficulties and greatly enhance the feasibility of research aiming to understand this interplay between determinism and neutrality^4^. Indeed, the study of microbial biogeography was effectively launched by the statement of Becking^5^, in 1934, that “everything is everywhere, but, the environment selects”. The culture-based approaches used at Becking's time may have biased the observations that lead to his statement, but recently developed techniques now allow microbial ecologists to observe the distributions of the vast majority of unculturable microorganisms and have subsequently revitalized research into this question, particularly in the last decade. However, despite decades of debate surrounding observations that microbial communities vary along gradients of soil physical and chemical properties^5^,^6^,^7^,^8^, the explicit importance of this environmental variation relative to dispersal limitation, stochastic demographics, and limiting similarity during microbial community assembly is still unclear^9^,^10^, particularly among divergent groups of microorganisms, limiting our ability to predict microbial community shifts and their functional consequences^11^,^12^, which are substantial^13^,^14^,^15^,^16^.

The vast majority of our knowledge regarding the biogeography of soil microbial communities is based on the sampling of topsoil (typically the top 10 or 30 cm) where the impacts of management (for example, tillage, fertilisation, compaction) are more likely to be experienced. The vertical structure of soil is influenced both by parent material weathering (bottom-up) and the organic inputs (top-down), potentially resulting in inconsistent and variable habitat turnover in space, providing an opportune gradient over which to test these processes. Topsoil communities are more likely to be influenced by large-scale dispersal of propagules (generally driven by the mass movement of air and water and aboveground fauna^17^), the relatively high density of roots^18^ and greater variation in soil temperature and moisture^19^. Immigration into deeper soil layers requires the activity of burrowing soil organisms and the vertical movement of water^20^,^21^. In addition, most microbial biogeography studies focus on bacterial communities^9^, but bacteria and fungi differ substantially in their growth habit (generally, unicellular versus filamentous growth) and dispersal capability (typically thought to be greater for bacteria and more variable across taxa for fungi due to differences in propagule size and number). Bacteria are also generally more resilient than fungi in the face of disturbance^22^ due to their relatively high intrinsic growth rates and unicellular nature, which will be particularly important in the upper soil layers where the impacts of management (for example, tillage, fertilisation, compaction) are more likely to be experienced. Climatic variation is also greater in the surface soil layers, and fungi and bacteria can vary in their response to minimal temperature for growth with fungi being more active at lower temperatures than bacteria^23^. Therefore, the outcomes of community assembly and their relation to vertical structure in soils may not be consistent between these two groups, which is important as bacteria and fungi in soils generally differ in their contributions to nutrient turnover and energy flow through soil food webs, with subsequent consequences for the rates and stability of element cycles^24^,^25^,^26^,^27^.

Here, our aim is to characterise the contributions of deterministic and stochastic processes to community assembly at large spatial scales and to contrast how ecologically dissimilar groups vary in the relative importance of these processes. We sampled soils from multiple horizons at 183 sites across Scotland, using a 20 × 20 km^2^ sampling grid. Each site included a central profile pit, to bedrock or 75 cm, and vertical subsamples were taken in each paedological horizon^28^, from which we sampled environmental DNA and characterised bacterial and fungal communities using DNA fingerprinting (terminal restriction fragment length polymorphism, T-RFLP). Community turnover is estimated from the strength of the distance decay relationship, as well as the relationship between community similarity and increasing community isolation in deeper soil horizons. To estimate the correspondence between microbial communities and environmental variables that may select for a subset of microbial taxa^29^, we also estimate habitat turnover within each soil horizon by measuring 51 edaphic properties relating to the chemical and physical characteristics of the soils collected from each horizon. We find that the power of habitat turnover to explain variation in bacterial and fungal community turnover is reduced in deeper soil layers. To test the hypothesis that increased community isolation due to dispersal limitation is behind the reduced explanatory power in deeper layers, we employ a powerful null model approach assuming that environmental variation is unimportant during community assembly, according to the neutral process as described by Hubbell^3^. To do this, we estimate immigration and dispersal parameters of the neutral model and use these estimates to simulate outcomes of neutral community assembly^30^,^31^. These simulated outcomes are used to estimate the central tendency and dispersion of distributions of pairwise similarities expected when neutral processes dominate community assembly and to calculate the effect sizes associated with deviations from this null hypothesis^32^,^33^. Finally, to evaluate whether these DNA fingerprinting data are appropriate for detecting the ecological patterns observed here, we also estimate effect sizes from a subset of samples for which bacterial communities are described using a high-throughput, DNA sequencing-based approach (454 pyrosequencing).

## Results

### Community and habitat turnover in space

**Figure 1:**
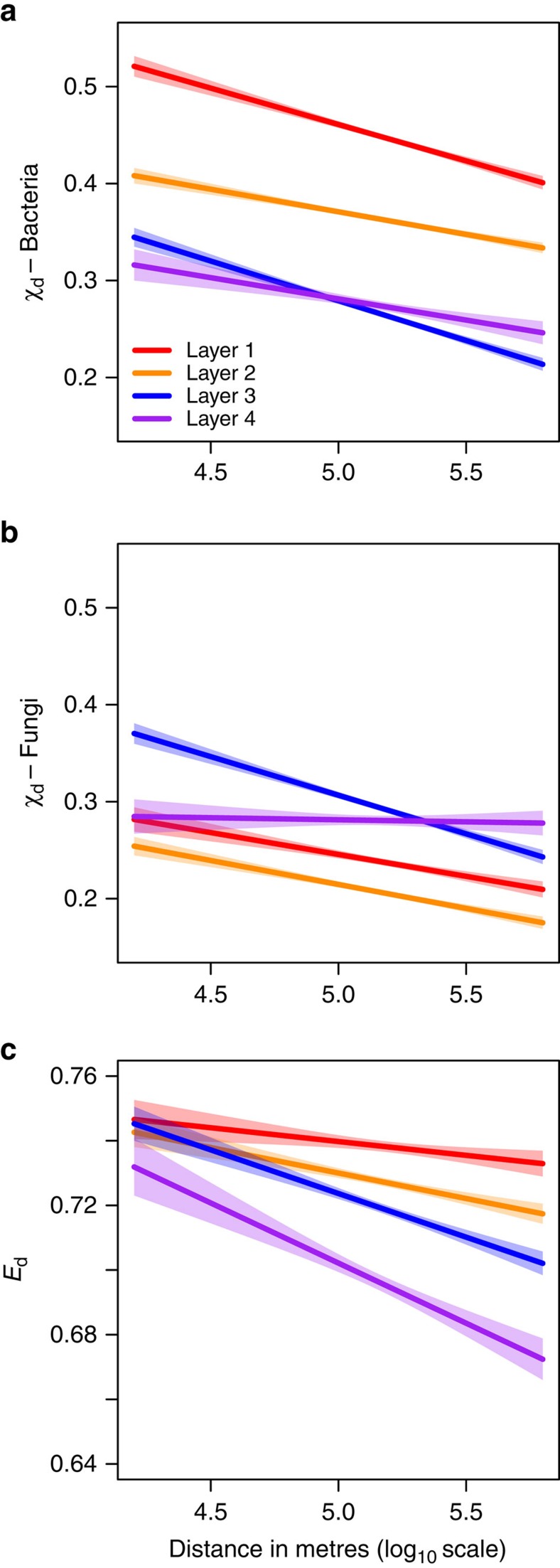
Community and habitat turnover with increasing geographic distance at the scale of Scotland. Pairwise community similarities (*X*_*d*_) are based on the Sørensen index for bacteria (**a**) and fungi (**b**) while pairwise habitat similarities (*E*_*d*_) are based on Euclidean distances (**c**). The shaded region represents the 95% confidence limits on the regression estimates. Model coefficients are provided in Supplementary Table 1; individual data points are plotted in Supplementary Fig. 1.

In general, for both bacteria and fungi, we observed that community turnover increased with increasing geographic distance at the scale of Scotland (Fig. 1 and Supplementary Fig. 1). However, bacterial and fungal communities exhibited different patterns in this relationship among soil layers. Pairwise community similarities for bacteria, on average, decreased in deeper soil layers indicating higher community turnover (Fig. 1a). The opposite pattern was observed for fungi and no significant relationship was observed between community similarity and geographic distance in the deepest soil layer (Fig. 1b). We did not find a significant association between bacterial and fungal community turnover (Sørensen community similarities; Mantel *r*=−0.015, *P*>0.1).

**Figure 2:**
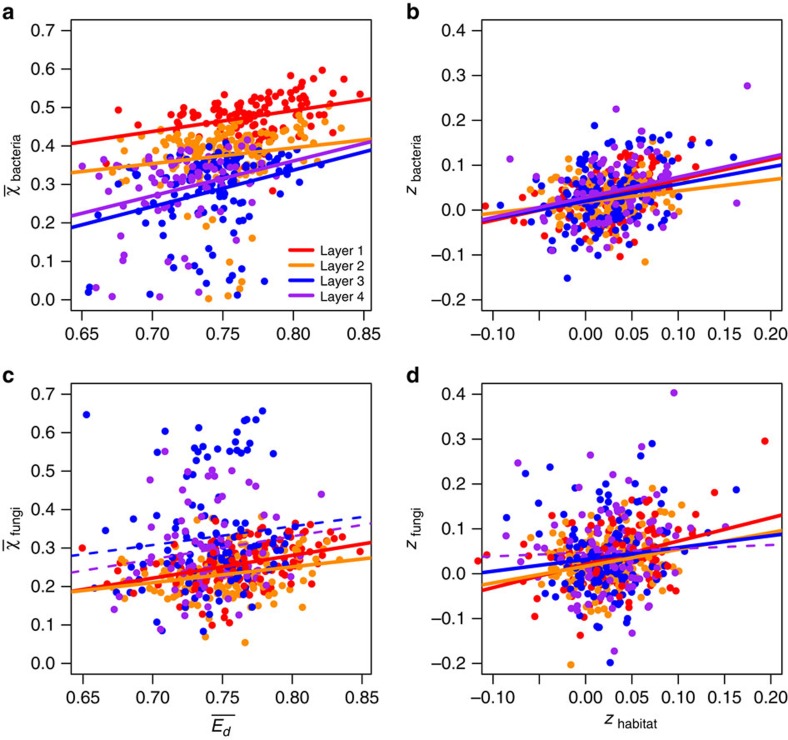
Relationships between habitat turnover and turnover of bacterial and fungal communities within neighbourhoods of 140 km. Two relationships are plotted for bacterial (**a**,**b**) and fungal (**c**,**d**) communities: the first compares mean levels of community (*χ*) and habitat (*E*_*d*_) similarity within each neighbourhood (**a**,**c**) and the second compares the strength of community/habitat turnover through space (*z*) within each neighbourhood (**b**,**d**). Lines indicate the predicted relationship, solid lines indicate the relationship is significant (*P*<0.05), dashed lines indicate the relationship is not significant based on type II linear regression estimated using ordinary least squares (*P*⩾0.05). *R*^2^⩾0.120 for the surface layer (layer 1) and *R*^2^≤0.096 for the deeper layers; model coefficients and individual *R*^2^-values are provided in Supplementary Table 5. Additional analyses were performed, including site-level variables (Supplementary Table 6) and on only a subset of the most important variables (Supplementary Fig. 2, Supplementary Table 7), revealing similar patterns in the relationships between community and habitat turnover.

Habitat similarity also decreased with increasing geographic distance and the strength of this relationship of habitat turnover with distance was greater in deeper soil layers. In addition, sample-to-sample variation in edaphic conditions in deeper soil layers was, on average, greater than in shallower soil layers (Fig. 1c). These observations suggest that the potential for environmental filtering in microbial communities should be greater for deeper soil layers. To test this, we estimated relationships between community turnover and habitat turnover (for distances up to a maximum of 140 km, at which autocorrelation was observed; see Supplementary Tables 2–4)^29^. The power of habitat turnover to explain variation in bacterial and fungal community turnover was reduced in deeper soil layers, both when looking at mean levels of turnover and the slope of community/habitat turnover through space (Fig. 2), even though we expected these relationships to increase in strength as habitat turnover increased.

### Neutrality as a model for community assembly

**Figure 3:**
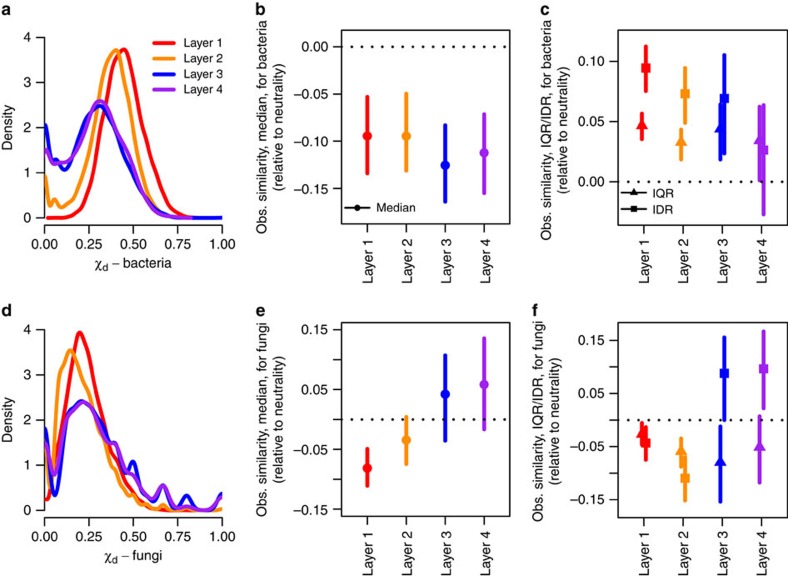
Distributions of pairwise similarities between microbial communities and effect sizes relative to a null model based on neutral community assembly. Community similarities (*X*_*d*_) for bacteria (**a**–**c**) and fungi (**d**–**f**) are based on the Sørensen index. Distributions of observed community similarities within each of the four soil layers are presented as probability densities (**a**,**d**). The mean (points) and 95% confidence interval (vertical lines) of the central tendency (**b**,**e**) and dispersion (**c**,**f**) of observed community similarities are presented relative to 100 simulations under the null model. Here, a reduction or increase in the central tendency is evidence of, in general, convergence upon or divergence from a common community composition, respectively. A reduction/increase in dispersion is further evidence of convergence/divergence, with a focus on extremes of the distribution; the interquartile range (IQR) and the interdecile range (IDR) provides estimates of dispersion in the middle 50 and 80% of the distribution, respectively.

Substantial variation was observed in the median but also in the dispersion of distributions in the calculated community similarities for bacteria and fungi across the soil horizons (Fig. 3a,d). The null model analysis indicated that there was strong evidence for bacterial communities demonstrating significant divergence (Fig. 3b,c), which is expected for niche-based assembly when the environment is heterogeneous^32^,^34^. Comparing patterns among soil layers, dispersion tended to resemble more closely the predictions under neutrality in deeper layers (Fig. 3c) suggesting a greater role for neutral assembly processes (that is, dispersal limitation and neutral drift) at depth and supported by the immigration parameter estimates from the neutral model being reduced in deeper layers (Table 1). However, taken as a whole, the evidence presented here suggests that bacterial assembly processes are still strongly dominated by the effects of niche differentiation, even in deeper soil layers.

In contrast, assembly processes in fungal communities were strongly and differentially affected across the different soil layers. In the topmost layer, the shift in the central tendency relative to the null model indicated divergence from a common community composition (Fig. 3e), again as expected in a heterogeneous environment. However, within this layer (and marginally in the next layer), we observed evidence of underdispersion in the distribution of effect sizes, suggesting that communities existing in similar habitats (where communities should converge upon a common structure) were more different than expected in the absence of niche differentiation (neutrality). Therefore, fungal communities were constrained to be highly dissimilar in a way that could not be explained by niche-based environmental filtering. In the two deeper soil layers, the confidence interval of the central tendency also overlapped with zero, suggesting an important role of neutral processes during community assembly (Fig. 2e). Examination of the estimates of dispersion in these layers revealed complex patterns due to subsets of communities converging upon a common composition (the peaks on the right of the distribution of Fig. 2d, representing pairs of communities with highly similar community composition) that is highly divergent from most other communities that were sampled.

### Influence of land use on community assembly processes

**Figure 4:**
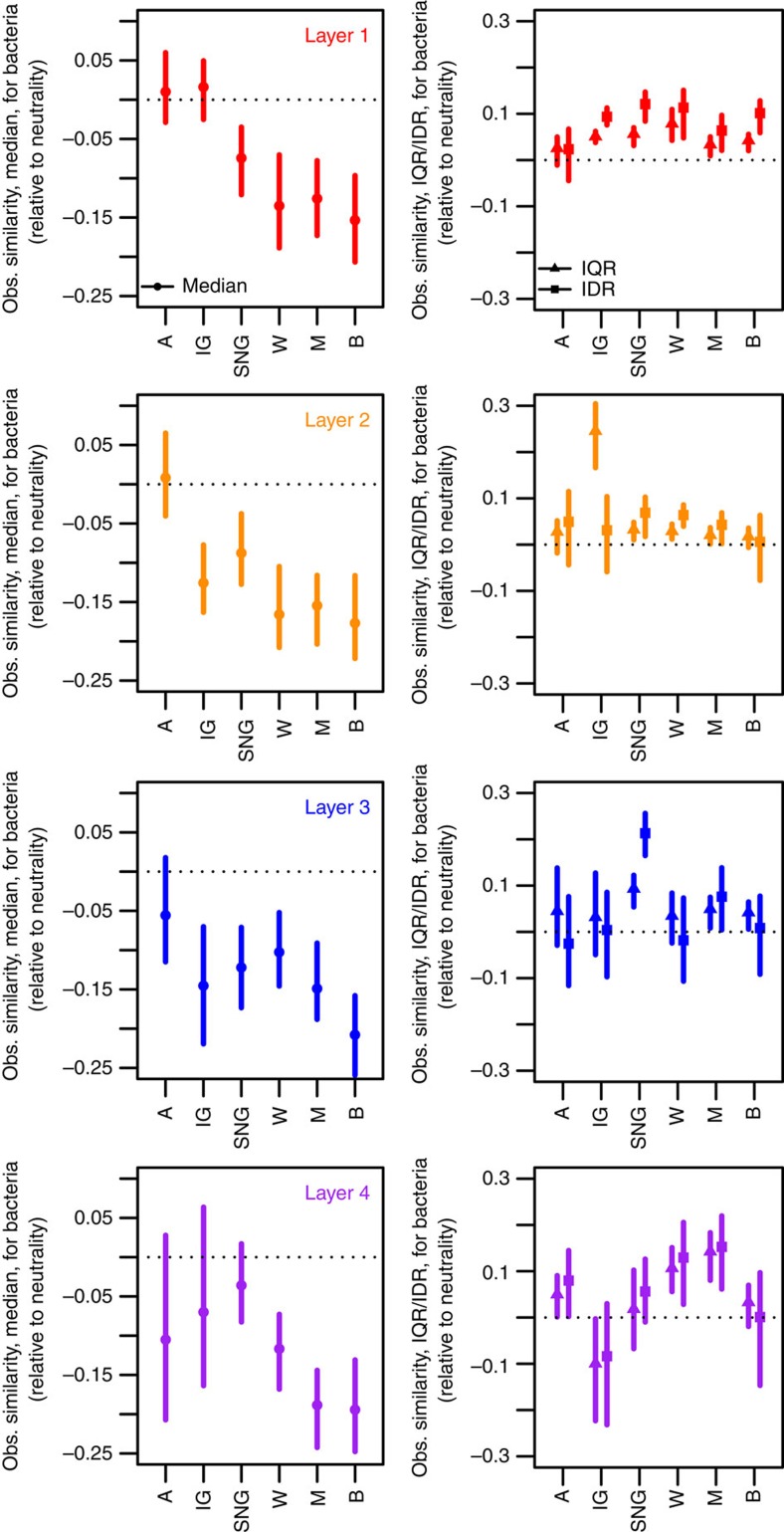
Effect sizes under different land uses relative to a null model based on neutral community assembly for bacteria sampled from four different soil layers. Estimates are associated with the central tendency (left) and dispersion (right) parameters of pairwise community similarities. A, arable; IG, improved grassland; SNG, semi-natural grassland; W, woodland; M, moor; B, bog. See Fig. 3 caption for details on how effect sizes were calculated.

**Figure 5:**
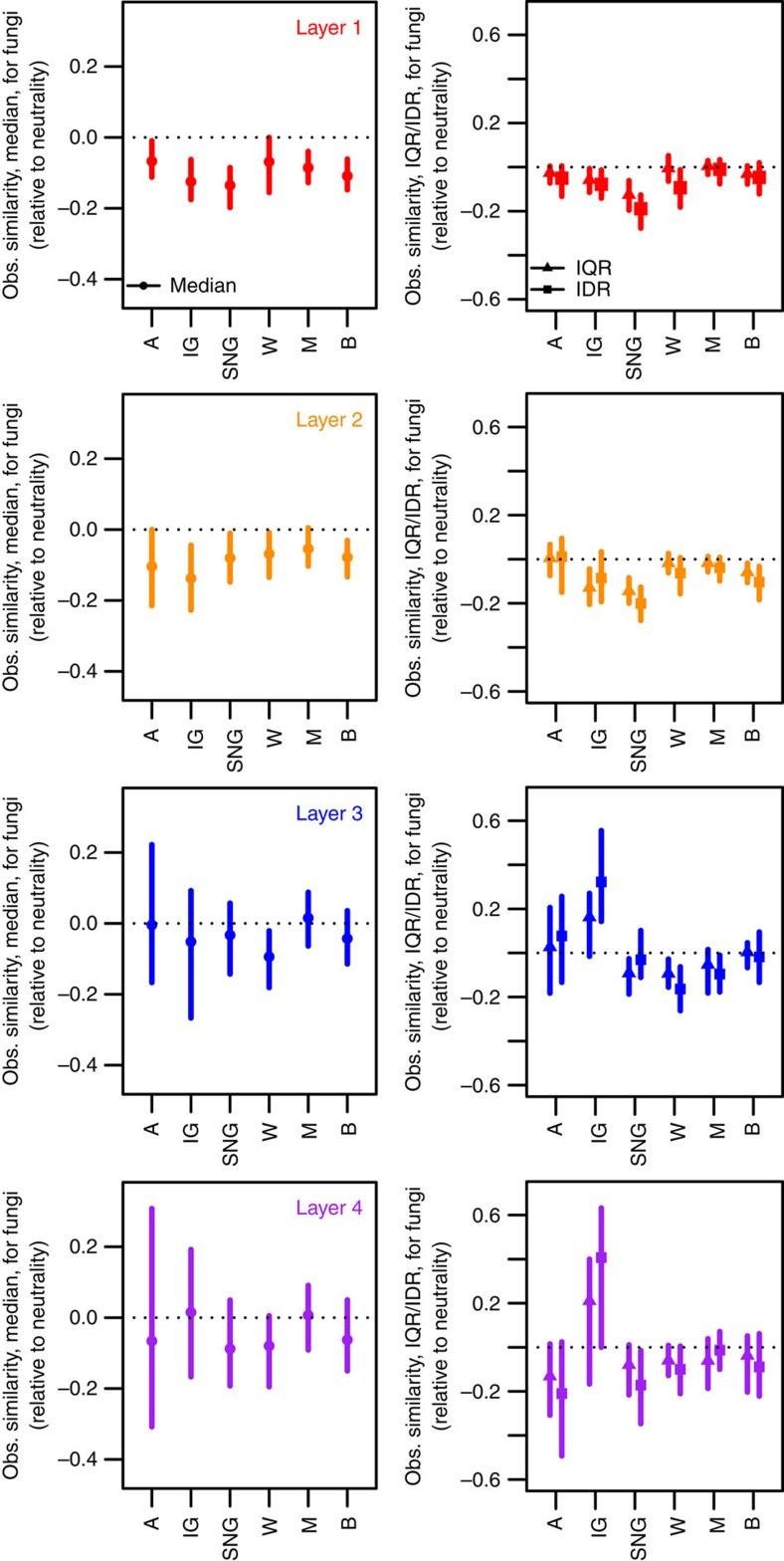
Effect sizes under different land uses relative to a null model based on neutral community assembly for fungi sampled from four different soil layers. Estimates are associated with the central tendency (left) and dispersion (right) parameters of pairwise community similarities. A, arable; IG, improved grassland; SNG, semi-natural grassland; W, woodland; M, moor; B, bog. See Fig. 3 caption for details on how effect sizes were calculated.

It has been suggested that a high level of disturbance and variation in the ability to recover from disturbance can lead to very high levels of community turnover^35^,^36^. Sampling took place across a variety of land-use intensities, which can be at least partly represented by the vegetation type observed at each sampling site (Supplementary Table 8), in that some sites experienced substantial manipulation (arable systems, improved grasslands) and several others are likely to have experienced some impact (semi-natural grasslands, woodlands). When we performed null model analysis within each category of land use (model parameter estimates in Supplementary Tables 9 and 10), we observed that bacterial communities demonstrated evidence of neutral processes increasing in importance during assembly under more intensive land use (especially arable land across all depths and for grasslands at certain depths) while exhibited patterns that were consistent with deterministic processes under reduced land-use intensity (some grasslands, particularly semi-natural grasslands, and under woodlands, moors and bogs; Fig. 4). Fungal communities, however, exhibited patterns suggesting that the relative importance of neutral and niche-based assembly was largely similar among the different levels of land use, regardless of what soil layers the fungal communities were sampled from (Fig. 5). The exception to this pattern is under improved grasslands at the two most isolated soil layers, where overdispersion was observed; this was the only signal of niche-based assembly in fungal communities and is the likely source of the complex patterns described above for the estimates of dispersion (Fig. 3f). Under all other land uses, the patterns in fungal communities were consistent with those predicted assuming neutral assembly or a highly stochastic assembly process.

### Comparing data types for detecting deviations from neutrality

The outcomes of null model analyses with high-throughput DNA sequencing and fingerprinting data were qualitatively similar, with both data types providing evidence for non-neutral dynamics. For both data types, estimates of the central tendency indicated that pairwise similarities among bacterial communities were reduced compared with neutral predictions, while distributions of pairwise similarities were overdispersed compared with neutral predictions (Supplementary Table 11). Where they differed, however, was in the magnitude of the deviation from the neutral expectation, with effect sizes being generally greater for bacterial communities characterised by pyrosequencing than when DNA fingerprints were used. This suggests that the lack of taxonomic resolution and associated noise inherent in DNA fingerprinting approaches may result in reduced power to detect non-neutral dynamics when sample sizes are small or along short environmental gradients. However, our conclusions are robust to this issue given that DNA fingerprinting revealed non-neutral dynamics for both bacteria and fungi, with the primary conclusion that the nature of these dynamics was substantially different between these broad taxonomic groups, especially with increasing dispersal limitation in deeper soil layers.

## Discussion

Various factors behind the differing contributions of environmental variation and isolation during bacterial and fungal community assembly may be at play, but all are mediated by differences between bacteria and fungi in dispersal capacity across the landscape and through the soil profile. The extreme stochasticity observed for fungi in surface layers may be due to strong antagonistic interactions that are governed primarily by priority effects^37^ (that is, organisms being more abundant within a patch due to their early arrival), but that dispersal limits the manifestation of these antagonistic interactions uniformly in the environment. Bacterial communities may also be subject to these priority effects^38^, but the manifestation of these effects will be less prevalent in the absence of dispersal limitation. Fungi in deeper soil layers may participate in fewer antagonistic interactions with other fungi. For instance, mycorrhizal fungi are more frequently observed in mineral soil layers than saprotrophic fungi^39^ and, while antagonistic interactions can occur among mycorrhizal fungi^40^, there is little evidence to suggest that these are as strong as interactions between saprotrophs that actively produce secondary metabolites with antagonistic properities^41^. Community turnover for bacteria was less predictable in deeper soil layers and influenced to a greater extent by neutral processes. For bacteria, passive dispersal through the soil profile is likely to be strongly negatively associated with propagule size^21^, increasing the role that dispersal limitation plays in assembly, but assembly outcomes were still strongly deterministic.

We have focussed on two very ecologically divergent taxonomic groups to assess the variable contributions of deterministic and stochastic processes to community assembly, but considerable ecological variation also exists within these taxa. For instance, propagule sizes vary substantially within each group and are typically within, but not exclusive to, the 5–50 μm diameter range for fungi^20^ and the 0.2–20 μm diameter range for bacteria^21^. Similarly, variation in resilience capacity exists within bacteria^42^ and fungi^43^ in their responses to stress and disturbance. Focussing further within these groups, with increasing resolution on taxa of particular ecological relevance, and utilising the analytical approaches described here will lead to further insight into the drivers of community assembly. Our data do not provide this insight as they were generated using a taxonomy-independent approach, but the use of next-generation sequencing techniques combined with novel trait-based approaches^44^,^45^,^46^ will aid these activities in the very near future.

In summary, we provide direct evidence supporting the hypothesis that the contributions of deterministic and stochastic assembly processes vary depending on the ecological context in which the processes are active and provide insight into the ecological characters involved in determining these contributions. The biogeography of soil bacteria is governed largely by habitat turnover, even when enhancing the potential for dispersal limitation to drive these effects. Fungal communities, on the other hand, demonstrated much clearer potential for dispersal limitation to influence the outcomes of community assembly via stochastic demographics and complex outcomes of biotic interactions. The high levels of dissimilarity observed, especially in upper horizons, represent a significant barrier to predicting how fungal community structure and composition are likely to respond to global environmental change, requiring dedicated research efforts^47^ but with intensive sampling at relevant spatial scales. A focused but novel framework on the study of interactions occurring within microbial communities, including manipulation of the soil environment but also the development of inoculation strategies, is necessary to take full advantage of their contributions to key ecosystem services.

## Methods

### Soil sampling

The location of sites and protocols for soil sampling and profile description are based on the National Soils Inventory of Scotland, 1978–1987, with soil characterisation and sampling protocols given in Lilly *et al*.^48^. Sampling for the current study was conducted in 2006–2009 as part of the resampling of the National Soils Inventory of Scotland (NSIS2; Supplementary Fig. 3) and the design is given in Chapman *et al*.^28^. Soil samples were collected from 183 sites across Scotland, using a 20 × 20 km^2^ sampling grid. Each site included a central profile pit from which a full description of soil characteristics to bedrock or 75 cm was collected and subsamples taken in each paedological horizon. Sample numbers and major soil groups associated with each horizon are presented in Supplementary Table 12. Field moist soils were sieved to <4 mm and visible pieces of plant material and soil animals were removed before use. Each sample was then separated into three subsamples and each treated in the following manner: (i) assayed for N mineralisation and NH_4_^+^ and NO_3_^−^ concentrations, (ii) air-dried for remaining chemical analyses and (iii) stored at −20 °C until DNA extractions could be performed. Methods associated with these analyses are described below.

### Microbial community analyses

DNA was extracted from ∼500 mg of soil samples using a modified^49^ protocol for the FastDNA SPIN kit for soil (Bio101, Vista, CA, USA), as described by Yao *et al*.^50^. Variation in soil chemical and physical properties can influence the quantity and quality of DNA extracts^51^; however, our data suggest that any bias associated with variation in these soil properties did not bias our ecological interpretations as bacterial and fungal DNA was amplified from the same DNA extracts and demonstrated divergent community patterns. These patterns in bacterial and fungal communities were characterised using multiplex T-RFLP^52^. T-RFLP profiles were obtained from 63F (VIC-labelled) and 1087R primers for bacteria and ITS1F (6-FAM-labelled) and ITS4 primers for fungi (primer sequences provided in Supplementary Table 13). The labelled PCR amplicons were checked by agarose gel electrophoresis and purified using a charge switch kit (Invitrogen). Approximately 500 ng of cleaned-up PCR multiplex product was digested with restriction enzyme *HhaI* (Promega); 2 μl of the digested DNA was mixed with 0.3 μl of GeneScan 500 LIZ dye size standard and 12 μl of Hi-Di formamide (both Applied Biosystems, Warrington, UK). Fragment size analysis was carried out with an ABI PRISM 3130xl genetic analyser (Applied Biosystems). Fragment analysis was performed using fragments sized between 35 and 550 bp. Relative abundance of terminal restriction fragments was calculated in T-REX software^53^ with clustering threshold of 0.99. Fragments with fluorescence units <50 and peaks with heights that were <2% of the total peak height were excluded from further analysis. These bacterial and fungal terminal restriction fragment tables are available in the Dryad Digital Repository ( http://dx.doi.org/10.5061/dryad.r3sh7).

While there is considerable debate regarding the use of community fingerprinting methods for analyses of microbial communities, these approaches can perform just as well as deep sequencing when investigating ecological patterns in microbial communities at multiple scales^54^. To compare the outcomes of null model analysis using fingerprinting data to those obtained through deep sequencing, we obtained a bacterial community matrix for the topsoil layer at 108 sites based on the output of 454 pyrosequencing of bacterial 16S rRNA. Pyrosequencing was performed on a Roche GS FLX System using a Titanium kit. A 466-bp fragment of 16S rRNA gene was amplified using the modified primers PRK341F and PRK806R (primer sequences provided in Supplementary Table 13). Barcode, linker primer and reverse primer sequences were removed from the raw sequence reads using the ‘split_libraries.py' script while setting minimum sequence length of 200 and minimum quality score of 20. The ‘Acacia' algorithm was used with default options to remove pyrosequencing noise^55^. Potential chimeras were removed using the UCHIME chimera detection (*denovo* mode) utility of the USEARCH v6.0.307 tool^56^. Similar sequences were binned into operational taxonomic units (OTUs) using the ‘UCLUST' method (minimum pairwise identity of 97%). The OTU-sample matrix was obtained by using ‘Quantitative Insights Into Microbial Ecology' (QIIME v 1.6.0) software package^57^. These data represent a subset of a larger data set that is currently being prepared for publication; the data used here are provided as Supplementary Data 1.

### Habitat characterisation

All the soil and environmental properties were obtained from the NSIS2 database. The database is in the process of being made publicly available by the James Hutton Institute via The Scottish Soils Database & Website project; the data used here are provided in Supplementary Data 2. Only chemical and physical characteristics that were measured on individual soil samples were included in the estimation of habitat turnover, including 51 soil properties (pH, organic C, total N and P, soil C/N ratio, moisture, NH_4_^+^ and NO_3_^−^ concentrations, soil N mineralisation, soil loss-on-ignition; sand, silt, clay; oxalate extractable P, Fe and Al, Mn; aqua regia-extracted elements namely Ag, As, Ba, Cd, Co, Cr, Cu, Hg, Mo, Ni, Pb, Pt, Se, Sr, Zn, Al, B, Ca, Fe, K, Mg, Mn, Na, P, S, Ti; exchangeable Na, K, Ca, Mg, Mn, Fe, Al and H). Descriptions of the methods used to obtain these data are described in Chapman *et al*.^28^ and Yao *et al*.^50^ Although additional data were collected from each of the sites (including altitude, slope, drainage, temperature, precipitation and maximum rooting depth)^58^, we chose to only focus on variables that were measured on individual soil samples (that is, from each horizon at each site) for the analyses in the main manuscript. Our analyses of these additional data demonstrated that this decision had very little effect on the estimation of habitat turnover and no effect on the inferences made from the relationship between habitat and community turnover (Supplementary Tables 5 and 6).

### Estimation of spatial dependence

In order to characterise the spatial dependence of bacteria, fungi and environmental habitat, the first five principal components of respective depth layers were modelled via a geostatistical approach. Spatially correlated random effect terms were modelled via spherical models except for PC 5 of the layer 1 of environmental data, which was modelled via exponential model^59^. Estimation of spatially correlated random effect terms were carried out using restricted maximum likelihood estimation algorithm. More details on estimation of spatially correlated random effect terms using restricted maximum likelihood estimation algorithm is given by Lark *et al*.^60^. Once the spatial model parameters were estimated, spatial dependence was calculated as a ratio between nugget variance and sill variance and expressed as a percentage. Nugget/sill ratio explains the proportion of spatially unstructured variation in relation to the total variation and lower values indicate strong spatial dependence while higher values indicate low spatial dependence. Cambardella *et al*.^61^ reported that nugget/sill ratio<25% indicate a strong spatial dependence (structure), while 25 to 75% and >75% indicate moderate and weak spatial dependence respectively. Model fitting was carried out using geoR^62^ package in R statistical programming language^63^.

### Estimation of community and habitat turnover

We used the approach described in Ranjard *et al*.^29^, with some modifications, to estimate the strength of community and habitat turnover across the whole of Scotland and to investigate the relationship between community and habitat turnover at spatial scales for which autocorrelation was observed. Briefly, pairwise bacterial and fungal community similarities were calculated based on the Sørensen index, using the labdsv package^64^ in R^63^, while habitat similarities were calculated from the Euclidean distance between sites (‘dist' function in R) based on edaphic properties using the formula


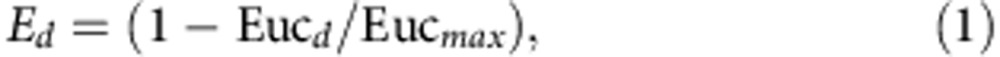


where Euc_*d*_ is the Euclidean distance function and Euc_*max*_ is the maximum distance between sites in the matrix. The slope of the distance decay relationship for bacterial and fungal communities was estimated using weighted linear regression and according to the formula


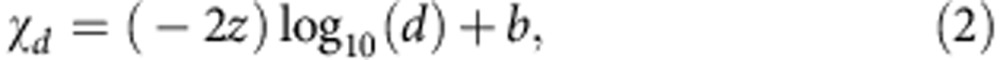


where *χ*_*d*_ represents the Sørensen index, *z* represents the turnover rate among communities, *d* represents the distance between sites in metres, and *b* represents the intercept of the relationship. The slope of the distance decay relationship for habitat was estimated by substituting *χ*_*d*_ for *E*_*d*_ in this formula. This procedure was performed across the entire data set and then repeated for each individual sampling point using a neighbourhood of 140 km to estimate (i) the relationship between mean community and habitat similarity and (ii) the relationship between *z*_habitat_ and *z*_bacteria_ or *z*_fungi_. Within each neighbourhood, we used the lmodel2 package^65^ in R to perform type II linear regression (ordinary least squares) since all parameters were estimated with errors.

To ensure that these observations were not due to the inclusion of environmental variables of little importance to microbial community composition, masking an existing relationship, we also repeated these analyses using the ten most important environmental predictors of bacterial or fungal community composition (separately, following forward selection of variables during constrained analyses of principal coordinates; Supplementary Fig. 2) while providing estimates of variance explained by each of these ten predictors using permutational multivariate analysis of variance (Supplementary Table 14); these analyses were conducted using the vegan package^66^ in R^63^. The results (Supplementary Table 7) were largely consistent with the outcomes of the analyses with the full environmental matrix (Supplementary Table 5).

### Null model analysis of microbial communities

We employed a null model approach utilising the neutral model of metacommunity and local community dynamics developed by Hubbell^3^. Parameters of the neutral model were estimated from the community data for bacteria and fungi after generating customised PARI/GP input files based on the example files provided by Etienne^31^. Parameters were initially estimated separately for bacteria and fungal communities and for each soil layer (that is, eight independent metacommunities), and then again after further separating these communities into groups representing each category of land use (that is, 48 independent metacommunities). We then used these parameter estimates to generate PARI/GP input files to simulate 1,000 communities of equal size under the assumption of neutral assembly, using the algorithm of Etienne^30^. Output files were imported into R^63^ to calculate pairwise similarities (Sørensen index^29^) among all communities within each simulation to generate distributions of the central tendency (median) and dispersion (interquartile range, interdecile range) of calculated similarities across all simulations, as well as confidence intervals for these estimates^32^,^33^. Effect sizes and their confidence intervals represent the difference between the estimate (median, interquartile range or interdecile range) from the distribution of observed pairwise similarities and the mean value of that same estimate from the simulated distributions; confidence intervals were generated using the estimate from the 2.5th and 97.5th percentile of the simulated distribtions^32^,^33^. R scripts for generating PARI/GP input files, reading output files, and estimating effect sizes are available at https://bitbucket.org/jrpowell/neutralnullmodels_r2gp.

For null model analysis within land-use categories, we used vegetation classifications aggregated into six major vegetation types: arable, improved grassland, semi-natural grassland, woodland, moorland and bog. Vegetation type is only a rough proxy for land-use intensity, as there are more intensive aspects of land use that were not accounted for in the available data (for example, historical fertiliser use and grazing). However, by analysing the data within these broader categories we are able to generate more robust comparisons of the distribution of community similarities and neutral predictions than if we were to do so for many categories with few samples collected within each. A summary of the sample numbers among these vegetation types and soil layers is provided in Supplementary Table 8, while a summary of sample numbers associated with major soil groups within each vegetation class and soil layer is provided in Supplementary Table 12.

To compare the outcomes of null model analysis using fingerprinting data to those obtained through deep sequencing, we then applied the null model approach to the OTU-sample matrix derived from 454 pyrosequencing data and to the OTU-sample matrix derived from the T-RFLP data obtained from these same samples. These analyses were performed across all samples and then again for samples within each vegetation type (except for woodlands since there was only one sample in this class).

## Additional information

**Accession codes:** Bacterial and fungal T-RFLP data have been deposited in the Dryad Digital Repository (http://dx.doi.org/10.5061/dryad.r3sh7). R scripts for generating PARI/GP input files, reading output files, and estimating effect sizes are available at https://bitbucket.org/jrpowell/neutralnullmodels_r2gp.

**How to cite this article:** Powell, J. R. *et al*. Deterministic processes vary during community assembly for ecologically dissimilar taxa. *Nat. Commun.* 6:8444 doi: 10.1038/ncomms9444 (2015).

## Supplementary Material

Supplementary Information Supplementary Figures 1-3, Supplementary Tables 1-14 and Supplementary References

Supplementary Data 1 Relative abundances of bacterial OTUs generated from 454 sequencing.

Supplementary Data 2 Environmental variables used in the analysis of bacterial and fungal community turnover. Descriptions of the variables can be found in the 'Habitat characterisation' section of the 'Methods' section (and publications cited within).

## Figures and Tables

**Table 1:** Estimated parameters associated with the neutral model of biodiversity fit at the level of Scotland for bacterial and fungal communities described within each soil layer.

**Response matrix**	**Soil layer**	**Theta**	**I (median)**	**I (IQR)**
Bacteria	1	34.68	18.60	12.25
	2	40.57	14.43	10.67
	3	40.51	10.12	9.37
	4	44.46	11.88	11.02
Fungi	1	54.79	7.71	8.74
	2	36.13	2.87	4.91
	3	16.49	1.10	2.59
	4	17.47	0.80	2.17

I, immigration; IQR, interquartile range; theta, biodiversity.

Theta is estimated for the entire metacommunity while I is estimated for each local community; the central tendency and dispersion of the distribution across communities is represented by the median and IQR, respectively.
