# Supplementary material for: Deterministic processes vary during community assembly for ecologically dissimilar taxa
Source: Nat Commun. 2015 Oct 5;6:8444. doi: 10.1038/ncomms9444 (PMC4600744; doi:10.1038/ncomms9444)
Supplement: Supplementary Information — Supplementary Figures 1-3, Supplementary Tables 1-14 and Supplementary References [file ncomms9444-s1.pdf]

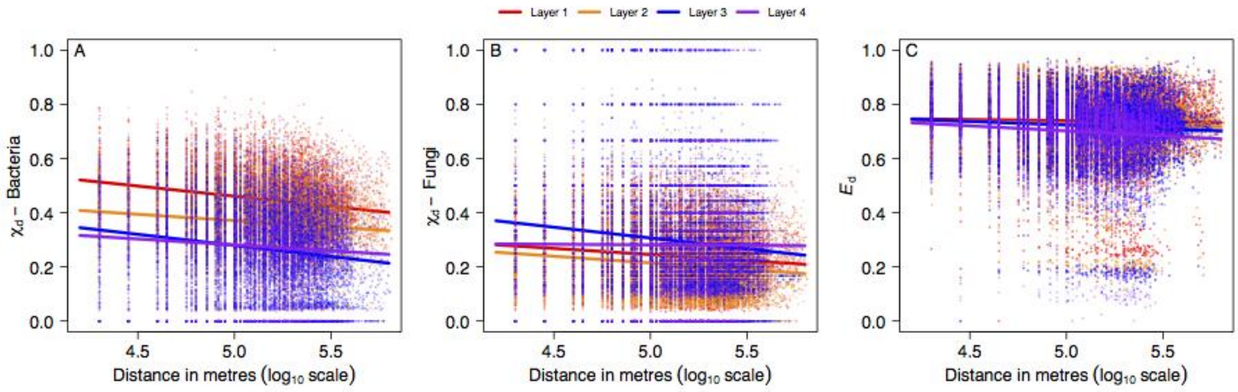

Supplementary Figure 1. Community and habitat turnover with increasing geographic distance at the scale of Scotland. Pairwise community similarities (*X_d*) are based on the Sorensen index for bacteria (A) and fungi (B) while pairwise habitat similarities (*E_d*) are based on Euclidean distances (C). Distances and linear relationships within each soil layer are indicated by colour, and the shaded region represents the 95% confidence limits on the regression estimates. Model coefficients are provided in Supplementary Table 1.

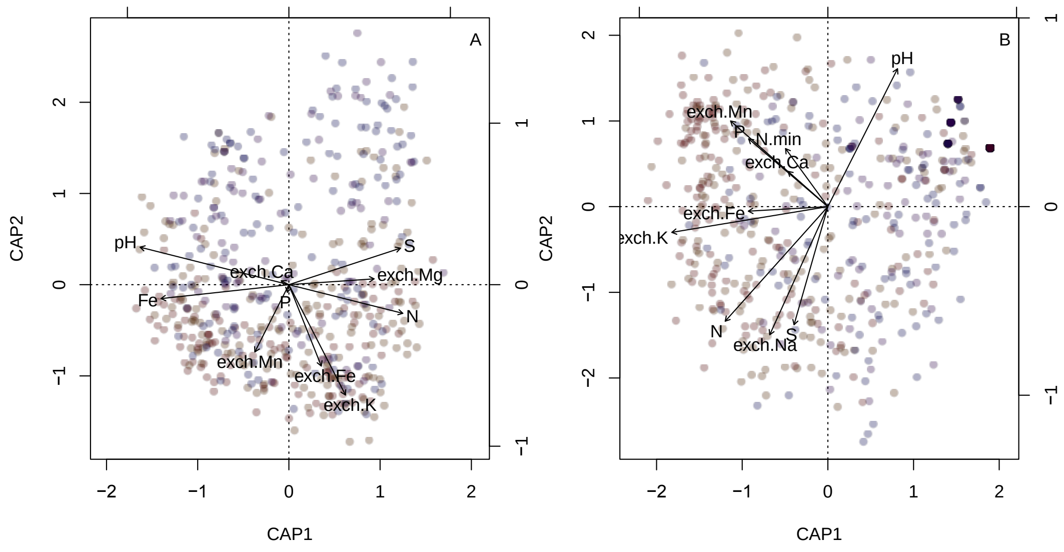

Supplementary Figure 2. Constrained analyses of principal coordinates for bacterial (A) and fungal (B) communities, using the top ten most important environmental variables for predicting community composition for each microbial group. Colours refer to the soil layers from which the communities were sampled (see Supplementary Fig. 1). The ten constraining variables pictured in each panel explained 13.2 % and 12.9 % of variation in bacterial and fungal communities, respectively. For comparison, using all 51 environmental variables that were measured on individual soil samples explained 22.6 % and 21.2 % of variation, while including additional site variables (altitude, slope, drainage, temperature, precipitation, and maximum rooting depth) explained 32.0 % and 29.9 % of variation in bacterial and fungal communities, respectively.

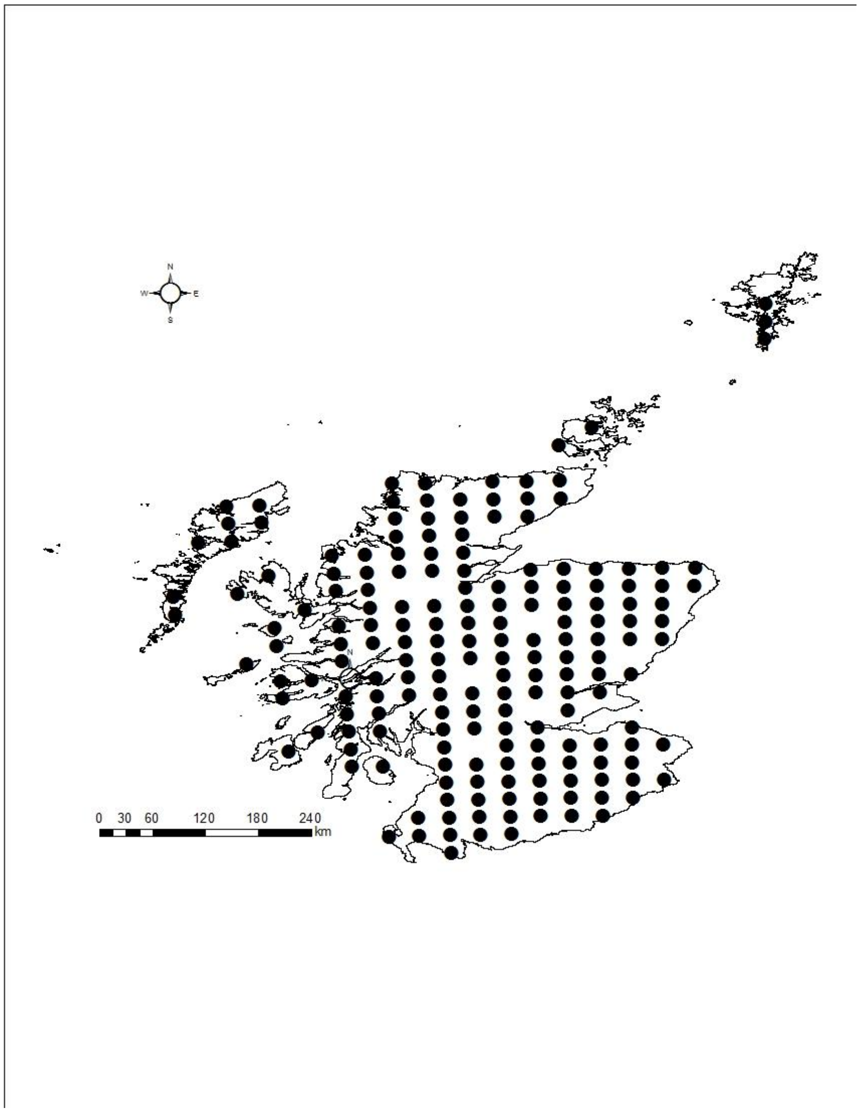

Supplementary Figure 3. Locations of sampling points distributed along a 20km grid across the whole of Scotland. More detailed maps, including land use categories, can be found in Yao et al.[6].

Supplementary Table 1. Estimated model parameters associated with community and habitat turnover at the scale of Scotland. The intercept reflects the mean pairwise similarity and *z* reflects the strength of the TAR/HAR pictured in Figure 1. Parameters were estimated and *P*-values calculated based on type II linear regression estimated using ordinary least squares. ****P* < 0.001, ***P* < 0.01, ns: non-significant.

Response matrix	Soil layer	Intercept	Lower CI	Upper CI	Slope	Lower CI	Upper CI	*P*-value
Bacteria	L1	0.448	0.446	0.450	0.038	0.034	0.041	***
	L2	0.363	0.361	0.365	0.023	0.020	0.027	***
	L3	0.265	0.262	0.268	0.041	0.035	0.047	***
	L4	0.274	0.268	0.279	0.022	0.012	0.031	***
Fungi	L1	0.238	0.236	0.240	0.023	0.018	0.027	***
	L2	0.206	0.204	0.208	0.025	0.021	0.028	***
	L3	0.293	0.289	0.297	0.040	0.033	0.047	***
	L4	0.281	0.274	0.287	0.002	-0.009	0.013	ns
Environment	L1	0.738	0.737	0.740	0.004	0.001	0.007	**
	L2	0.727	0.726	0.729	0.008	0.006	0.010	***
	L3	0.719	0.718	0.721	0.013	0.011	0.016	***
	L4	0.696	0.693	0.699	0.019	0.013	0.024	***

Supplementary Table 2: Estimated spatially correlated random effect terms for fungal communities.

Layer	PC	Nugget	Sill	Nugget/Sill*100	Range (m)
1	1	0.003	0.008	37.50	200000
	2	0.005	0.008	62.50	200000
	3	0.005	0.007	71.43	200000
	4	0.005	0.008	62.50	100000
	5	0.005	0.008	62.50	20000
2	1	0.004	0.006	66.67	200000
	2	0.003	0.006	50.00	200000
	3	0.005	0.006	83.33	100000
	4	0.005	0.006	83.33	200000
	5	0.006	0.006	100.00	237093
3	1	0.002	0.004	50.00	50000
	2	0.002	0.008	25.00	200000
	3	0.004	0.006	66.67	100000
	4	0.005	0.006	83.33	50000
	5	0.005	0.006	83.33	50000
4	1	0.055	0.06	91.67	150000
	2	0.022	0.039	56.41	100000
	3	0.034	0.036	94.44	64968
	4	0.027	0.032	84.38	150000
	5	0.022	0.027	81.48	100000

Supplementary Table 3: Estimated spatially correlated random effect terms for bacterial communities.

Layer	PC	Nugget	Sill	Nugget/Sill*100	Range (m)
1	1	0.004	0.008	50.00	200000
	2	0.005	0.007	71.43	200000
	3	0.005	0.008	62.50	50000
	4	0.005	0.008	62.50	100000
	5	0.006	0.008	75.00	50000
2	1	0.005	0.006	83.33	150000
	2	0.004	0.006	66.67	200000
	3	0.005	0.006	83.33	150000
	4	0.005	0.006	83.33	50000
	5	0.005	0.006	83.33	100000
3	1	0.005	0.007	71.43	100000
	2	0.005	0.007	71.43	100000
	3	0.005	0.007	71.43	50000
	4	0.004	0.007	57.14	50000
	5	0.005	0.007	71.43	50000
4	1	0.008	0.013	61.54	150000
	2	0.009	0.012	75.00	50000
	3	0.009	0.012	75.00	50000
	4	0.007	0.012	58.33	50000
	5	0.01	0.012	83.33	50000

Supplementary Table 4: Estimated spatially correlated random effect terms for environmental variables.

Layer	PC	Nugget	Sill	Nugget/Sill*100	Range (m)
1	1	0.12	0.282	42.55	200000
	2	0.12	0.166	72.29	200000
	3	0.07	0.25	28.00	50000
	4	0.08	0.355	22.54	200000
	5	0.22	0.232	94.83	149787
2	1	0.19	0.303	62.71	150000
	2	0.13	0.245	53.06	50000
	3	0.16	0.302	52.98	150000
	4	0.08	0.314	25.48	200000
	5	0.22	0.267	82.40	100000
3	1	0.16	0.353	45.33	150000
	2	0.19	0.262	72.52	200000
	3	0.19	0.349	54.44	150000
	4	0.07	0.367	19.07	200000
	5	0.21	0.25	84.00	50000
4	1	0.11	0.242	45.45	150000
	2	0.12	0.254	47.24	100000
	3	0.14	0.434	32.26	150000
	4	0.04	0.561	7.13	200000
	5	0	0.284	0.00	28843

Supplementary Table 5. Estimated model parameters associated with relationships between community turnover and habitat turnover. Habitat turnover was estimated from the full set of 51 environmental variables measured independently for each soil layer. Two relationships are presented: the first compares mean levels of community (χ) and habitat (E_d) similarity within each neighbourhood and the second compares the strength of the TAR and HAR (z) within each neighbourhood. Parameters were estimated and *P*-values calculated based on type II linear regression estimated using ordinary least squares.

Response matrix	Relationship	Soil layer	Intercept	Lower CI	Upper CI	Slope	Lower CI	Upper CI	*P*-value	R^2
Bacteria	$\chi_{community}$ ~ E_d	L1	0.056	-0.155	0.267	0.545	0.272	0.819	0.001	0.120
		L2	0.067	-0.239	0.373	0.410	0.003	0.818	0.029	0.025
		L3	-0.421	-0.802	- 0.040	0.948	0.436	1.460	0.001	0.096
		L4	-0.365	-0.872	0.141	0.908	0.205	1.611	0.007	0.081
	$z_{community}$ ~ $z_{habitat}$	L1	0.022	0.013	0.031	0.452	0.259	0.646	0.001	0.144
		L2	0.018	0.010	0.025	0.247	0.064	0.431	0.006	0.041
		L3	0.020	0.005	0.035	0.381	0.066	0.696	0.008	0.040
		L4	0.028	0.012	0.045	0.450	0.146	0.753	0.001	0.095
Fungi	$\chi_{community}$ ~ E_d	L1	-0.214	-0.432	0.004	0.618	0.335	0.901	0.001	0.138
		L2	-0.093	-0.282	0.096	0.430	0.178	0.682	0.002	0.068
		L3	0.023	-0.498	0.544	0.406	-0.292	1.104	0.144	0.009
		L4	-0.047	-0.639	0.546	0.447	-0.357	1.250	0.134	0.015
	$z_{community}$ ~ $z_{habitat}$	L1	0.020	0.009	0.030	0.442	0.235	0.648	0.001	0.122
		L2	0.017	0.007	0.027	0.397	0.162	0.631	0.002	0.061
		L3	0.031	0.015	0.046	0.358	-0.002	0.719	0.026	0.024
		L4	0.046	0.020	0.073	0.144	-0.350	0.639	0.278	0.004

Supplementary Table 6: Estimated model parameters associated with relationships between community turnover and habitat turnover. Habitat turnover was estimated from all environmental variables measured independently for each soil layer and categorical data collected at the site level (altitude, slope, drainage, temperature, precipitation, and maximum rooting depth). Two relationships are presented: the first compares mean levels of community (χ) and habitat (E_d) similarity within each neighbourhood and the second compares the strength of the TAR and HAR (z) within each neighbourhood. Parameters were estimated and *P*-values calculated based on type II linear regression estimated using ordinary least squares.

Response matrix	Relationship	Soil layer	Intercept	Lower CI	Upper CI	Slope	Lower CI	Upper CI	*P*-value	R^2
Bacteria	$\chi_{community} \sim E_d$	L1	0.118	-0.074	0.309	0.468	0.218	0.718	0.001	0.107
		L2	0.134	-0.168	0.436	0.322	-0.081	0.725	0.061	0.016
		L3	-0.249	-0.631	0.132	0.718	0.207	1.230	0.006	0.058
		L4	-0.431	-0.948	0.087	0.996	0.279	1.713	0.004	0.094
	$z_{community} \sim z_{habitat}$	L1	0.022	0.013	0.031	0.471	0.281	0.662	0.001	0.159
		L2	0.019	0.011	0.026	0.227	0.045	0.410	0.006	0.035
		L3	0.021	0.006	0.035	0.372	0.053	0.691	0.013	0.037
		L4	0.029	0.012	0.046	0.446	0.138	0.754	0.002	0.091
Fungi	$\chi_{community} \sim E_d$	L1	-0.267	-0.480	-0.053	0.686	0.408	0.964	0.001	0.169
		L2	-0.132	-0.322	0.059	0.479	0.226	0.732	0.001	0.081
		L3	0.051	-0.461	0.562	0.368	-0.316	1.052	0.153	0.008
		L4	0.130	-0.412	0.673	0.209	-0.525	0.944	0.277	0.004
	$z_{community} \sim z_{habitat}$	L1	0.019	0.009	0.029	0.456	0.250	0.661	0.001	0.130
		L2	0.017	0.008	0.027	0.393	0.159	0.627	0.002	0.061
		L3	0.030	0.014	0.045	0.391	0.035	0.746	0.019	0.030
		L4	0.046	0.020	0.072	0.160	-0.331	0.652	0.277	0.005

Supplementary Table 7: Estimated model parameters associated with relationships between community turnover and habitat turnover. Habitat turnover was estimated from the ten most important environmental variables, measured independently for each soil layer, for explaining variation in bacterial or fungal community composition (analysed separately, Supplementary Fig. 2). Two relationships are presented: the first compares mean levels of community (χ) and habitat (E_d) similarity within each neighbourhood and the second compares the strength of the TAR and HAR (z) within each neighbourhood. Parameters were estimated and *P*-values calculated based on type II linear regression estimated using ordinary least squares.

Response matrix	Relationship	Soil layer	Intercept	Lower CI	Upper CI	Slope	Lower CI	Upper CI	*P*-value	R^2
Bacteria	$\chi_{community} \sim E_d$	L1	0.231	0.139	0.323	0.418	0.261	0.574	0.001	0.188
		L2	0.204	0.047	0.361	0.271	0.025	0.517	0.026	0.028
		L3	0.158	-0.015	0.330	0.227	-0.075	0.529	0.071	0.017
		L4	0.119	-0.113	0.352	0.302	-0.117	0.720	0.088	0.026
	$z_{community} \sim z_{habitat}$	L1	0.020	0.012	0.028	0.445	0.307	0.584	0.001	0.242
		L2	0.014	0.007	0.020	0.484	0.323	0.645	0.001	0.174
		L3	0.011	-0.002	0.025	0.623	0.379	0.867	0.001	0.156
		L4	0.040	0.024	0.057	0.063	-0.199	0.325	0.296	0.003
Fungi	$\chi_{community} \sim E_d$	L1	-0.016	-0.117	0.085	0.449	0.285	0.612	0.001	0.191
		L2	0.040	-0.071	0.152	0.275	0.112	0.438	0.001	0.062
		L3	0.148	-0.102	0.397	0.275	-0.110	0.661	0.089	0.013
		L4	0.286	0.060	0.511	0.003	-0.385	0.390	0.467	0.000
	$z_{community} \sim z_{habitat}$	L1	0.017	0.007	0.027	0.391	0.237	0.545	0.001	0.163
		L2	0.012	0.003	0.021	0.681	0.424	0.938	0.001	0.138
		L3	0.027	0.012	0.041	0.666	0.321	1.011	0.001	0.086
		L4	0.040	0.017	0.063	0.466	0.069	0.864	0.014	0.056

Supplementary Table 8: Number of samples associated with each land use category

Response matrix	Layer	Arable	Improved Grassland	Semi-natural Grassland	Woodland	Moorland	Bog
Bacteria	1	17	28	22	10	32	23
	2	14	26	30	29	37	35
	3	10	16	26	27	30	33
	4	3	7	15	22	22	16
Fungi	1	17	28	22	11	32	24
	2	15	27	31	29	38	35
	3	12	25	29	27	32	32
	4	3	12	16	24	24	15

Supplementary Table 9. Estimated parameters associated with the neutral model of biodiversity, fit at the level of Scotland, for bacterial communities described within each soil layer and within each land use category.

Land use	Soil layer	*theta*	*I* (median)	*I* (IQR)
Arable	1	35.76	27.40	10.10
	2	56.62	19.30	5.49
	3	33.57	1.37	24.00
	4	148.33	6.60	1.63
Improved Grassland	1	38.80	25.08	4.31
	2	36.49	22.42	19.59
	3	38.62	10.19	18.91
	4	29.60	3.66	7.43
Semi-natural Grassland	1	34.26	18.62	5.01
	2	40.79	16.21	8.75
	3	45.16	10.97	6.72
	4	53.02	13.07	9.80
Woodland	1	23.84	17.23	3.05
	2	22.21	16.46	7.11
	3	33.59	14.37	13.79
	4	39.69	14.46	11.07
Moorland	1	24.06	21.76	12.74
	2	27.71	19.30	11.23
	3	32.28	14.73	10.99
	4	37.44	19.39	12.49
Bog	1	21.99	27.00	11.80
	2	24.65	18.91	15.67
	3	22.16	13.91	7.94
	4	26.09	11.62	9.07

Supplementary Table 10. Estimated parameters associated with the neutral model of biodiversity, fit at the level of Scotland, for fungal communities described within each soil layer and within each land use category.

Land use	Soil layer	*theta*	*I* (median)	*I* (IQR)
Arable	1	33.80	14.04	10.43
	2	13.02	2.03	3.04
	3	4.55	0.21	0.42
	4	4.49	1.75	2.93
Improved Grassland	1	26.61	12.00	18.60
	2	8.26	2.60	4.44
	3	2.70	0.26	0.54
	4	2.10	0.31	0.52
Semi-natural Grassland	1	30.96	8.27	15.77
	2	18.69	3.16	7.12
	3	8.35	1.12	3.76
	4	5.26	1.09	3.68
Woodland	1	49.23	4.59	4.05
	2	33.79	4.89	4.78
	3	14.58	2.99	2.91
	4	13.57	1.75	5.19
Moorland	1	51.39	7.82	7.97
	2	33.93	4.53	5.18
	3	16.02	1.43	2.60
	4	12.30	0.91	4.08
Bog	1	44.11	10.46	9.93
	2	30.45	3.13	5.73
	3	12.71	1.59	1.92
	4	9.81	1.39	2.74

Supplementary Table 11. Effect sizes associated with all samples and samples associates with specific vegetation types relative to a null model based on neutral community assembly for bacteria sampled from topsoil layers and characterised using 454 pyrosequencing or DNA fingerprinting (T-RFLP). The mean and 95% confidence interval of the central tendency (median) and dispersion (interquartile range; IQR) of observed community similarities are presented relative to 100 simulations under the null model.

Samples	Pairwise comparisons (samples)	Data type	Effect size (median)	Lower CI	Upper CI	Effect size (IQR)	Lower CI	Upper CI
All	5778	454	-0.273	-0.288	-0.250	0.189	0.179	0.197
	(108)	T-RFLP	-0.091	-0.128	-0.050	0.053	0.042	0.061
Arable	136	454	-0.232	-0.255	-0.206	0.068	0.059	0.075
	(17)	T-RFLP	-0.007	-0.047	0.038	0.024	-0.003	0.049
Improved Grassland	378	454	-0.227	-0.245	-0.205	0.114	0.108	0.119
	(28)	T-RFLP	-0.002	-0.041	0.036	0.053	0.037	0.063
Semi-natural Grassland	210	454	-0.275	-0.295	-0.249	0.076	0.052	0.098
	(21)	T-RFLP	-0.043	-0.088	0.004	0.064	0.042	0.079
Moorland	325	454	-0.366	-0.388	-0.342	0.065	0.054	0.071
	(26)	T-RFLP	-0.122	-0.171	-0.074	0.034	0.015	0.052
Bog	91	454	-0.329	-0.356	-0.300	0.086	0.077	0.095
	(14)	T-RFLP	-0.156	-0.209	-0.106	0.050	0.028	0.072

Supplementary Table 12. Number of samples associated with each soil type (major soil groups) based on land use category.

Land use	Soil layer	Alluvial soils	Brown soils	Gley soils	Peat	Podzols	Rankers	Regosols
Arable	1	2	8	10	0	14	0	0
	2	2	8	7	0	12	0	0
	3	2	6	6	0	8	0	0
	4	0	0	2	0	4	0	0
Improved Grassland	1	6	30	8	0	10	2	0
	2	6	29	8	0	10	0	0
	3	6	23	6	0	6	0	0
	4	4	9	4	0	2	0	0
Semi-natural Grassland	1	2	2	18	6	10	4	2
	2	2	4	28	6	14	5	2
	3	2	3	24	6	12	6	2
	4	0	2	16	2	9	0	2
Woodland	1	0	4	2	6	8	1	0
	2	0	10	8	14	22	4	0
	3	0	9	8	14	21	2	0
	4	0	6	8	10	22	0	0
Moorland	1	0	0	20	16	20	8	0
	2	0	0	21	18	30	6	0
	3	0	0	18	16	28	0	0
	4	0	0	14	12	20	0	0
Bog	1	0	0	2	45	0	0	0
	2	0	0	4	66	0	0	0
	3	0	0	4	61	0	0	0
	4	0	0	2	29	0	0	0

Supplementary Table 13. PCR primers used in this study.

Primer name	Primer sequence	Reference
63F	AGGCCTAACACATGCAAGTC	Marchesi et al.[1]
1087R	CTCGTTGCGGGACTTAACCC	Hauben et al.[2]
ITS1F	CTTGGTCATTTAGAGGAAGTAA	Gardes and Bruns[3]
ITS4	TCCTCCGCTTATTGATATGC	White et al.[4]
PRK341F	CCTAYGGGRBGCASCAG	Cai et al.[5]
PRK806R	GGACTACNNGGGTATCTAAT	Cai et al.[5]

Supplementary Table 14. Permutational multivariate analysis of variance results for the ten most important environmental variables, measured independently for each soil layer, for explaining variation in (separately) bacterial and fungal community composition. Terms were entered in the model in the order indicated by forward selection on all environmental variables.

Response matrix	Predictor	R^2	Cumulative adjusted R^2	Pseudo-*F* statistic	*P*-value
Bacteria	pH	0.065	0.063	41.7	0.001
	exch.K	0.029	0.091	18.5	0.001
	exch.Mn	0.013	0.102	8.5	0.001
	exch.Mg	0.029	0.130	18.8	0.001
	N	0.010	0.139	6.5	0.001
	Fe	0.012	0.149	7.7	0.001
	exch.Ca	0.007	0.155	4.6	0.001
	S	0.014	0.166	8.7	0.001
	P	0.006	0.171	3.6	0.001
	exch.Fe	0.005	0.174	3.1	0.001
Fungi	pH	0.041	0.039	27.2	0.001
	exch.Mn	0.037	0.075	24.5	0.001
	exch.Na	0.016	0.088	10.6	0.001
	exch.K	0.034	0.121	22.4	0.001
	N	0.013	0.132	8.4	0.001
	P	0.007	0.137	4.4	0.001
	exch.Ca	0.008	0.143	5.4	0.001
	exch.Fe	0.005	0.147	3.3	0.001
	S	0.009	0.155	6.3	0.001
	N.min	0.008	0.162	5.3	0.001

Supplementary References

1. Marchesi, J. R. *et al.* Design and evaluation of useful bacterium-specific PCR primers that amplify genes coding for bacterial 16s rRNA. *Appl. Environ. Microbiol.* **64,** 795–799 (1998).
2. Hauben, L., Vauterin, L., Swings, J. & Moore, E. R. Comparison of 16S ribosomal DNA sequences of all *Xanthomonas* species. *Int. J. Syst. Bacteriol.* **47,** 328–335 (1997).
3. Gardes, M. & Bruns, T. D. ITS primers with enhanced specificity for basidiomycetes - application to the identification of mycorrhizae and rusts. *Mol. Ecol.* **2,** 113–118 (1993).
4. White, T. J., Bruns, T., Lee, S. & Taylor, J. W. Amplification and direct sequencing of fungal ribosomal RNA genes for phylogenetics. Pp 315-322 in *PCR Protocols: A Guide to Methods and Applications* (Academic Press, Inc., 1990).
5. Cai, L., Ye, L., Tong, A. H. Y., Lok, S. & Zhang, T. Biased diversity metrics revealed by bacterial 16S pyrotags derived from different primer sets. *PLoS ONE* **8,** e53649 (2013).
6. Yao, H. *et al.* Multi-factorial drivers of ammonia oxidizer communities: evidence from a national soil survey. *Environ. Microbiol.* **15,** 2545–2556 (2013).
